# Targeting Rac and Cdc42 GEFs in Metastatic Cancer

**DOI:** 10.3389/fcell.2020.00201

**Published:** 2020-04-08

**Authors:** Maria del Mar Maldonado, Julia Isabel Medina, Luis Velazquez, Suranganie Dharmawardhane

**Affiliations:** Department of Biochemistry, School of Medicine, University of Puerto Rico Medical Sciences Campus, San Juan, Puerto Rico

**Keywords:** Rac, Cdc42, guanine nucleotide exchange factors, metastasis therapy, guanine nucleotide exchange factor (GEF), metastasis, targeted (selective) treatment

## Abstract

The Rho family GTPases Rho, Rac, and Cdc42 have emerged as key players in cancer metastasis, due to their essential roles in regulating cell division and actin cytoskeletal rearrangements; and thus, cell growth, migration/invasion, polarity, and adhesion. This review will focus on the close homologs Rac and Cdc42, which have been established as drivers of metastasis and therapy resistance in multiple cancer types. Rac and Cdc42 are often dysregulated in cancer due to hyperactivation by guanine nucleotide exchange factors (GEFs), belonging to both the diffuse B-cell lymphoma (Dbl) and dedicator of cytokinesis (DOCK) families. Rac/Cdc42 GEFs are activated by a myriad of oncogenic cell surface receptors, such as growth factor receptors, G-protein coupled receptors, cytokine receptors, and integrins; consequently, a number of Rac/Cdc42 GEFs have been implicated in metastatic cancer. Hence, inhibiting GEF-mediated Rac/Cdc42 activation represents a promising strategy for targeted metastatic cancer therapy. Herein, we focus on the role of oncogenic Rac/Cdc42 GEFs and discuss the recent advancements in the development of Rac and Cdc42 GEF-interacting inhibitors as targeted therapy for metastatic cancer, as well as their potential for overcoming cancer therapy resistance.

## Introduction

**FIGURE 1:**
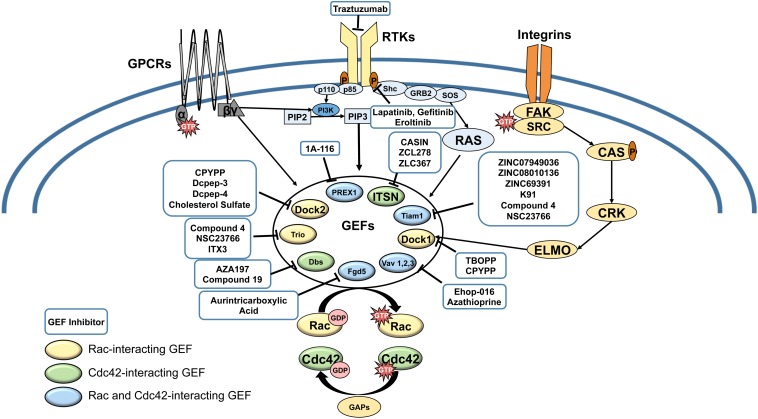
Receptor tyrosine kinases (RTKs), G-protein coupled receptors (GPCRs) and Integrin pathways converge in the activation of Rac and Cdc42 GEFs. Cell surface receptors activate GEFs by a variety of mechanisms, involving phosphoinside 3-kinase (PI3-K)-mediated production of phosphatidyl-inositol (3,4,5) tris phosphate (PIP3), which activates GEFs such as P-Rex1, DOCKs, and Vavs; Ras, which activates Tiam-1; and Focal adhesion kinase (FAK)/ signaling to activate ELMO a partner for DOCK1. Upregulation of Rac and Cdc42 can lead to resistance to cell surface receptor targeted therapies (eg. Trastuzumab, Lapatinib, Gefitinib, Eroltinib). Rac/Cdc42-interaction inhibitors are shown in blue boxes. Color-coding of GEFs represent Rac-exclusive GEFs, Cdc42-exclusive GEFs, and GEFs that interact with both Rac and Cdc42.

Rho GTPases have emerged as pivotal drivers of cancer metastasis in multiple cancer types. Of the Rho GTPases, Rac (1,2,3) and the close homolog Cdc42 are central regulators of cancer cell migration and invasion, and thus, metastasis. Therefore, recent studies have focused on demonstrating the dysregulation of Rac and Cdc42 in cancer, understanding their contribution to the hallmarks of metastasis: cancer cell growth, cell cycle progression, survival, epithelial to mesenchymal transition (EMT), cell-cell and cell-substrate adhesion, cell polarization, vesicle trafficking, angiogenesis, immune function, and migration/invasion, and consequently, therapy resistance. Even though oncogenic point mutations and splice variants have been reported from a number of cancers, Rac and Cdc42 do not have to be mutated in cancer to drive cancer progression. This is due to their activation by a number of oncogenic cell surface receptors, including receptor tyrosine kinases (RTKs), G protein coupled receptors (GPCR), cytokine receptors, and integrin receptors, as well as Wnt and Notch signaling, which converge on Rac and Cdc42 by activating specific guanine nucleotide exchange factors (GEFs) (Figure 1). As culled from the Cancer Genome Atlas (TCGA) data, many GEFs themselves are oncogenic and are overexpressed or mutated in a number of cancer types (Table 1). Therefore, Rac and Cdc42 activation, even in the absence of oncogenic mutations or dysregulated expression, can play a critical role in cancer progression to metastasis.

**TABLE 1:** Percentage (%) alterations (*amplification, **mutations, ***deep deletion, ****multiple alterations) in GEFs of multiple cancers from the TCGA PanCancer Atlas (Cerami et al., 2012; Gao et al., 2013).

Type of Cancer	Tiam-1	Trio	P-Rex1	P-Rex2	Vav1	Vav2	Vav3	Dock1	Ect2
Esophageal Squamous Cell Carcinoma	**2.11%	*16.8%	**3.16%	**1.05% *3.16%	**1.05% ***1.05%	*2.11%	0%	**1.05% *1.05%	*28.42%
Ovarian Epithelial Tumor	***1.2%	**1.54% *7.02%	**1.71% *2.91%	**1.2% *5.99%	*1.03%	0%	0%	*2.74%	*23.12%
Non-small cell lung cancer	**7.69%	*11.02% **5.13%	**6.17% *1.04%	**6.74% *1.99%	**2.37%	**1.8%	**3.8%	**1.99%	*19.37%
Head and Neck Squamous Cell Carcinoma	**2.29%	**3.44% *3.63%	**2.49%	**3.25% *2.68%	**1.34%	0%	0%	**2.68%	*16.73%
Undifferentiated Stomach Adenocarcinoma	**15.38%	**7.69%	*7.69% **7.69%	***7.69%	0%	0%	0%	**7.69%	0%
Endometrial Carcinoma	**10.75%	**13.31%	**6.83% *2.05%	**7.51% *2.56%	**5.8%	**5.63%	**5.63%	**11.77%	**4.27% *7.34%
Cervical adenocarcinoma	**4.35%	**2.17%	*2.17%	**2.17%	***2.17%	**4.35%	**4.35%	**2.17%	*8.7% ****2.17
Esophagogastric Adenocarcinoma	**6.61%	**8.17% *4.28%	**4.09% *3.11%	**12.26%	**2.72%	**2.14%	**2.92%	**3.89% *1.36%	**1.17% *5.84%
Pancreatic Adenocarcinoma	**1.63%	0%	**2.72%	**1.09% * 3.8%	**1.63%	0%	0%	**1.63%	**2.17% * 2.72%
Invasive Breast Cancer	0%	**1.38% *2.4%	**1.11%	**2.03% *5.17%	0%	0%	0%	**1.11%	*2.58%
Colorectal Adenocarcinoma	**6.06%	**5.89%	**4.88% *6.57%	**6.73% *2.19%	**2.53%	**1.85%	**3.03%	**4.21%	**2.53%
Prostate Adenocarcinoma	0%	**1.21%	*1.01%	*6.48%	0%	*1.21%	**1.01%	0%	*2.43%
Glioblastoma	0%	**1.52%	**1.52%	0%	**1.18%	0%	0%	**1.18%	*1.01%
Melanoma	**9.01%	**10.14% ***1.13	**5.41% ***1.13%	**22.52% ***1.35%	**8.11%	**3.60%	*1.35% **5.41%	**7.43%	**4.05%

The pivotal role of Rac and Cdc42 in multiple cancers has been extensively reviewed by us and others (Pai et al., 2010; Mack et al., 2011; Stengel and Zheng, 2011; Wertheimer et al., 2012; Bid et al., 2013; Kazanietz and Caloca, 2017; Casado-Medrano et al., 2018; Maldonado and Dharmawardhane, 2018; De et al., 2019). In addition, the related Rho GTPase Rho has also been implicated in tumorigenesis and cancer progression. Multiple recent reviews have discussed the relevance, especially of RhoA and RhoC signaling in cancer, and thus, all three GTPases Rho, Rac, and Cdc42 are potential targets for anticancer drug development (Orgaz et al., 2014; Aspenström, 2018; Svensmark and Brakebusch, 2019; Porazinski et al., 2020). However, Rac and Cdc42 proteins share 72% sequence identity, and therefore, have a number of common upstream and downstream effectors such as p21-activated kinase (PAK), while Rho proteins activate different effectors such as Rho kinase (ROCK) and thus, activate distinct signaling cascades (Mott et al., 1999; Schmidt and Hall, 2002; Bustelo et al., 2007; Prudnikova et al., 2015). Therefore, even though all Rho family GTPases play a role in cancer malignancy, this review will focus on new strategies to target Rac and Cdc42 activation via blocking the exchange of GDP to GTP. We will also briefly discuss the burgeoning relevance of blocking Rac and Cdc42 to overcome cancer therapy resistance.

## Targeting Rac and Cdc42/GEF Interaction

The Rho GTPases have been considered “undruggable” for many years due to their limited availability of binding pockets and globular structure. The fact that they bind GTP with high affinity, and the micromolar concentrations of intracellular GTP, further complicates the development of GTP-competitive inhibitors. However, elucidation of the molecular basis for Rho GTPase activation by GEFs using crystal structure analysis coupled to functional mutant analysis of specific residues in Rac and Cdc42 that interact with different GEFs have enabled a deeper understanding of Rho GTPase/GEF molecular interactions. This knowledge in turn have fueled efforts to develop inhibitors that block the molecular interaction between GEFs and Rac and Cdc42. Herein, we discuss the relevance of Rac/Cdc42 GEFs in metastatic cancer cell signaling in the context of the GEF interaction inhibitors that target Rac and Cdc42 activation (Table 2).

**TABLE 2:** GEF-interaction inhibitors of Rac and Cdc42 in cancer.

Rac-GEF interaction inhibitors				

GEF	Inhibitor	References	IC50	*In vivo* models
Dock 1	TBOPP	Tajiri et al., 2017	8.4 μM	Lung metastasis mouse model
Dock 1, 2, and 5	CPYPP	Nishikimi et al., 2012	∼ 23 μM	–
Dock 2	Dcpep-3	Sakamoto et al., 2017	12 nM	–
	Dcpep-4	Sakamoto et al., 2017	6 nM	–
	Cholesterol sulfate	Sakurai et al., 2018	2 μM	–
Dock 5	C21	Vives et al., 2011	n.d	Bone metastasis mouse model (Vives et al., 2015)
Fgd5	Aurintricarboxylic acid	Park et al., 2019	157 nM	–
p-Rex	1A-116	Cardama et al., 2014a, b	1–10 μM	Experimental metastasis mouse model
Tiam	ZINC07949036	Ferri et al., 2009	24.1 μM	–
	ZINC08010136	Ferri et al., 2009	12.2 μM	–
	ZINC69391	Cardama et al., 2014a, b	31–61 μM	Breast cancer metastasis mouse model
	K91	Niebel et al., 2013	Kd = 209 nM	–
Tiam 1, Trio, Vav2	Compound 4	Ferri et al., 2013a	8.7 μM	–
Tiam-1, Trio	NSC23766	Gao et al., 2004	50–100 μM	Anaplastic large-cell lymphoma (ALCL)- Mice/xenograft model (Colomba et al., 2011) Chronic myelogenous leukemia mice model (Thomas et al., 2007) Murine orthopic gliobastoma xenograft model (Karpel-Massler et al., 2013)
TrioN	ITX3	Bouquier et al., 2009	50-100 μM	−
Vav	EHop-016	Montalvo-Ortiz et al., 2012	1 μM	Metastatic breast cancer mouse model (Castillo-Pichardo et al., 2014) Myxofibrosarcoma xenografts in mice (Okada et al., 2017)
	Azathioprine	Poppe et al., 2006	n.d	Pancreatic cancer mouse model (Razidlo et al., 2015)

** Cdc42-GEF interaction inhibitors**				

**GEF**	**Inhibitor**	**References**		

Dbs	AZA197	Zins et al., 2013	1–10 μM	Xenograft model of colon cancer
	Compound 19	Cummings et al., 2009	∼67 μM	–
Intersectin	CASIN	Peterson et al., 2006	2 μM	Colorectal cancer Mice/xenograft model (Sakamori et al., 2014)
	ZCL278	Friesland et al., 2013	Kd = 11.4 μM	–
	ZLC367	Aguilar et al., 2019	0.098 nM	Xenograft mouse model of lung cancer
PIP-2 mediated GEF activity	Pirl1	Peterson et al., 2006	3 μM	–

Rac and Cdc42 are structural homologs of the Rho GTPase family that are activated by common and distinct GEFs. The ∼80 different Rac/Cdc42 GEFs, can be divided into two groups depending on their conserved active domains: the diffuse B cell lymphoma (Dbl) Homology (DH)/Pleckstrin Homology (PH) proteins and Dedicator of Cytokinesis (DOCK) proteins. Approximately 70 GEFs share DH/PH domains, while 11 GEFs belong to the DOCK family (Rossman et al., 2005). The DH domain is required for GEFs to interact with Rho GTPases and catalyze GDP/GTP exchange, while the PH domain is used to stabilize or anchor with lipids and proteins (Harian et al., 1994; Sondek et al., 2000; Hoffman and Cerione, 2002; Cook et al., 2014). The DOCK family GEFs share the DOCK Homology Region 2 (DHR2), and a phospholipid-binding domain (DHR1) (Meller et al., 2005). DOCK proteins catalyze their GEF activity through the DHR2 domain, while the DHR1 is used to localize to the membrane by interacting with phosphoinositides, such as phosphatidyl-inositol (3,4,5) tris phosphate (PIP3). DOCK family GEFs interact only with Rac and Cdc42 and not RhoA.

The expression of GEFs are dependent on different tissues and cellular functions (Rossman et al., 2005). In addition, GEFs are activated by a myriad of cell surface receptors and cytosolic proteins, thus, adding to the complexity of their regulation. In cancer, Rac1 and Cdc42 are mutated, overexpressed and (or) hyperactivated. Hyperactivation of Rac and Cdc42 is primarily due to signaling via oncogenic upstream receptors, which in turn stimulate GEF activity (Arias-Romero and Chernoff, 2013; Kazanietz and Caloca, 2017; Maldonado and Dharmawardhane, 2018; De et al., 2019). However, GEFs themselves can become oncogenic by mutation or dysregulated expression (Table 1). Overexpressed GEFs have been detected in several types of cancer and correlated with clinicopathological features like poor prognosis and low survival rates. The most common GEFs implicated in cancer are T-cell lymphoma invasion and metastasis 1 (Tiam1), Trio, Vav1/2/3, Ect2, Phosphatidylinositol 3,4,5-trisphosphate (PIP3)-dependent Rac exchanger 1 (P-Rex1), and Dock1 (Wertheimer et al., 2012; Porter et al., 2016; Kazanietz and Caloca, 2017). This review will summarize the current knowledge on the GEFs that contribute to cancer malignancy and discuss therapies targeting the interaction of oncogenic GEFs with Rac and Cdc42.

### Targeting Tiam1

Tiam1 is a ∼177 kD protein that was first identified from invasive variants of murine leukemia cells (Habets et al., 1994). Tiam1 has affinity for both Rac1 and Cdc42, but is specific for Rac1 and Rac2 activation (Jaiswal et al., 2013). Structurally, Tiam-1 is composed of a myristoylation sequence, coiled-coil region, PEST, N-terminal and C-terminal PH, extended, Ras binding, PSD-95/DlgA/ZO-1, and DH domains. Similar to other GEFs, Tiam1 is auto-inhibited by its N-terminal PH domain coiled-coiled extension, blocking its GEF activity (Xu Z. et al., 2017). Tiam1 is regulated through phosphorylation, which localizes Tiam1 to the membrane to interact with cell surface receptors (Boissier, 2013). The myristoylation sequence, coiled-coil region and extended domain of Tiam1 interacts with membrane associated proteins, such as Par3, which controls polarization via Rac1 activation (Chen and Macara, 2005; Nishimura et al., 2005; Boissier, 2013; Matsuzawa et al., 2016).

Several studies have identified TIAM1 as an oncogene due to its overexpression or mutation in epithelial cancers with poor prognosis, such as non-small cell lung cancer (NSCLC), stomach, endometrial, colorectal, pancreatic, and cervical cancer (Table 1). For instance, in colorectal cancer, Tiam1 overexpression was associated with resistance to late stage therapeutics FOLFOX (5-fluorouracil and oxaliplatin) and FOLFIRI (folfinic acid, fluorouracil and irinotecan) (Izumi et al., 2019). Moreover, in papillary thyroid cancer, esophageal squamous cell carcinoma, metastatic hepatocellular carcinoma, and cervical cancer, Tiam1 overexpression was implicated with poor prognosis and a low survival rate (Hsueh et al., 2011; Liu et al., 2011; Huang et al., 2013; Yang et al., 2018). In addition to overexpression, Tiam1 was shown to be mutated in neuroblastoma where certain point mutations were associated with a better outcome in patients with neuroblastoma, and thus, implicated with dysregulation in Tiam1 signaling (Sanmartín et al., 2017). In breast cancer, Tiam1 has been implicated with invasion and metastasis, where Tiam1 overexpression was associated with high-grade breast cancer (Li et al., 2016). Therefore, blocking the Tiam1/Rac1 interaction has the potential to reduce metastasis from multiple cancer types.

Resolution of the crystal structure of the DH/PH domain of Tiam1 with Rac1 facilitated the rational identification of specific inhibitors of this interaction Worthylake et al., 2000). Structure-based mutagenesis of specific residues in the β2 and β3 strands in the switch 1 (residues 30–48) and Switch II (residues 59–75) regions of Rac1 identified Trp56 as a residue that is critical for GEF binding to Rac (Gao et al., 2001; Karnoub et al., 2001). Based on this premise, the small molecule NSC23766 was identified through a structure-based virtual screen as an inhibitor of the Tiam1 and Trio GEF interactions with Rac1 (Gao et al., 2004). Even though its high IC_50_ (∼50 μM) limits its therapeutic use, NSC23766 reduces invasion and growth of multiple cancer types, including prostate, breast, gastric, leukemia, lymphoma, and glioblastoma (Gao et al., 2004; Thomas et al., 2007; Yoshida et al., 2010; Colomba et al., 2011; Karpel-Massler et al., 2013; Lee et al., 2015). Recent studies have also demonstrated the potential of NSC23766 in combination therapy to overcome resistance to targeted as well as cytotoxic therapies (Karpel-Massler et al., 2017; Tian et al., 2018). Nonetheless, although the use of NSC23766 may look promising in some instances, the high effective concentrations (*μ*M range) and the reported off-target effects in platelets, on alfa-1-adrenergic receptors and CXCR4 chemokine receptors, limits its therapeutic use (Dutting et al., 2015; Mills et al., 2018; Yu et al., 2019).

Other small molecule inhibitors of the Tiam-1/Rac1 interaction were developed by the Ferri et al. (2009, 2013a,b) and Ruffoni et al. (2014) group through a docking-based virtual library screening, also using the Trp56 residue as a target. Compounds ZINC08010136 (IC_50_, 12.2 *μ*M) and ZINC07949036 (IC_50_, 24.1 *μ*M), and ZINC69391 (IC_50_ of 61 *μ*M) are some of the molecules identified by this group as Rac1/Tiam1 inhibitors. In the case of ZINC69391, this molecule was able to inhibit cell cycle progression, cell proliferation, migration, and lung metastasis in highly invasive breast cancer models (Cardama et al., 2014a). Using the structure of ZINC69391 as a template, a more potent Rac inhibitor, named 1A-116, was produced, but this molecule has a different mechanism of action that does not involve Tiam-1.

Besides chemical compounds, nucleic acid aptamers can also target Tiam1-mediated activation of Rac. K91 is an RNA aptamer inhibitor of the Rac-Tiam1 interaction. This RNA inhibitor consists of a full-length sequence of 94 nucleotides, corresponding to a molecular mass of 31 kDa, which specifically binds to the DH/PH domain of Tiam-1 *in vitro* (KD = 209 ± 45 nM) (Niebel et al., 2013). However, the efficacy of K91 in cell-based and *in vivo* models remains unknown. Potential challenges of this therapeutic strategy include difficulties in targeting the delivery to cancer cells and instability *in vivo.*

### Targeting Trio

Trio is a related ∼350 kD protein of the Dbl family of GEFs that contain two GEF domains: the first N-terminal domain contains the catalytic activity for Rac1 and RhoG and the second domain for RhoA (Debant et al., 1996). Additionally, it contains a CRAL-TRIO/SEC14 domain, two SH3 domains, a kinase domain, immunoglobulin motif and several spectrum repeats (Cannet et al., 2014). Moreover, Trio contains approximately five isoforms, Trio A-E, as a result of alternative splicing and differing in the C-terminus. One of the oncogenic isoforms, Tgat, is detected in adult leukemia and is specific for RhoA (Yoshizuka et al., 2004). Trio is activated by oncogenic upstream receptors such as RTKs and GPCRs. For example, in uveal melanoma, prolonged signaling via GPRCs activated Rac1 through Trio to signal to the transcriptional co-activator YAP and induce tumorigenesis (Feng et al., 2014).

The extension of lamellipodia by Trio requires the N-terminal GEF domain to interact with the C-terminus of Rac1 (Rijssel et al., 2012). During cytokinesis, Trio controls F-actin remodeling via the Rac1-actin related protein (Arp)2/3 pathway (Cannet et al., 2014). Phosphorylation of Trio at tyrosine 2681 (Y2681) leads to the activation of Rac1 and promotes colorectal cancer invasion. Trio pY1990 and Trio pY2681 phosphorylations were also correlated with clinicopathological parameters, where patients with high levels of Trio pY2681 demonstrated reduced survival (Sonoshita et al., 2015). Studies have also reported Trio to regulate invadopodia via Rac1/PAK signaling, specifically in metastatic breast carcinoma (Moshfegh et al., 2014). Therefore, in breast cancer tissues, Trio levels, but not Tiam1 or Vav1, were high and correlated with a poor outcome (Lane et al., 2008). Studies have shown Trio overexpression and (or) mutations in esophageal squamous cell carcinoma, NSCLC, stomach cancer, endometrial cancer, colorectal cancer, and glioblastoma (Table 1) (Schmidt et al., 2014). In glioblastoma, patients with a low survival rate had higher expression of Trio, Ect2 and Vav3, as well as high Rac1 expression (Salhia et al., 2008).

The compounds NSC23766 and CPYPP also inhibit Trio and have been tested in a variety of cancer models. Another Trio inhibitor is the small molecule ITX3, which blocks Trio’s binding to Rac with high specificity; however, its low potency (IC_50_ ∼50–100 *μ*M) limits its therapeutic potential (Bouquier et al., 2009). Lastly, compound 4, one of the molecules identified by the Ferri et al. (2013a) group, also inhibits Trio-mediated activation of Rac in a concentration-dependent manner; nonetheless its action is not-specific to Trio, since it also inhibits the GEFs Vav and Tiam 1.

### Targeting Vav1/2/3

Vav family proteins Vav1, Vav2, and Vav3 are composed of zinc-finger, proline rich motif, calponin-homology, Acidic, DH, PH, SH2, and two SH3 domains. Vav1 and 2 have broad specificity and act as GEFs for Rho, Rac, and Cdc42 (Movilla et al., 2001); however, studies have shown that Vav2, which is expressed in non-hematopoietic tissues is specific for Rac1 activation (Jaiswal et al., 2013; Cook et al., 2014). The GEF activity of Vav proteins are activated through phosphorylation of the c-terminal tyrosine residues via kinases activated in response to GPCRs, RTKs, integrin, and T-cell and B-cell receptors (Aghazadeh et al., 2000; Moores et al., 2000; Bartolomé et al., 2007).

Vav1 is exclusively expressed in hematopoietic tissues but not non-hematopoietic normal tissues. However, studies have detected Vav1 expression in several types of cancer cells and tumor tissue, including neuroblastoma, ovarian, lung, pancreatic, head and neck, gastric, endometrial, cervical, and colorectal cancer, metastatic melanoma, and B-cell chronic lymphocytic leukemia (Cook et al., 2014; Farago et al., 2020) (Table 1). Vav1 knockdown reduced tumor growth in nude mice from NSCLC cells expressing oncogenic K-Ras (Lazer et al., 2009). Vav1 also regulated pancreatic cancer cell migration via Rac1 (Razidlo et al., 2013), and its expression was correlated with pancreatic cancer cell lines but not in the normal human pancreas. Moreover, demethylation of VAV1 in the promoter region was shown to increase Vav1 expression in pancreatic cancer, and targeting Vav1 inhibited pancreatic cancer metastasis (Fernandez-zapico et al., 2005; Razidlo et al., 2015). Similarly, VAV1 gene is hypomethylated in medulloblastoma, leading to increased expression of Vav1 protein, which was correlated with the proto-oncogene MYCN amplification (Lindsey et al., 2014). Therefore, there is a need to elucidate epigenetic mechanisms regulating Vav expression, which can lead to novel therapeutic strategies.

Vav1 is expressed in a majority of breast tumors; however, Vav1 may induce apoptosis in certain breast cancer cell lines, in a p53 dependent manner (Sebban et al., 2013). This effect may explain the observation that in early invasive breast cancer, high nuclear expression of Vav1 was correlated with a lower incidence of disease relapse (Grassilli et al., 2014). The E59K and D51E oncogenic Vav1 point mutations identified from human lung adenocarcinoma resulted in truncation and overexpression of Vav1 with increased catalytic GEF activity for Rac1 activation (Shalom et al., 2018). D797N, another Vav1 mutation, described from NIH-3T3 fibroblasts, also demonstrated enhanced activation of Rac1 and upregulated the downstream effector c-Jun, thereby confirming transformation properties to Vav1 (Razanadrakoto et al., 2015). Therefore, Vav1 may play opposing roles depending on the type of cancer, and further studies are needed to determine and differentiate the signaling axis by which Vav1 is expressed and activated in malignant tumors.

Vav2 and Vav3 homologs are expressed in non-hematopoietic tissues and have been implicated with cardiovascular diseases and cancer (Perretta-Tejedor et al., 2017). Vav2/3 has been shown to specifically activate Rac1 and Rac3, the isoforms expressed in non-hematopoietic cells (Jaiswal et al., 2013). Moreover, Vav2/3 overexpression and activation has been reported from multiple cancers, including metastatic melanoma, endometrial, cervical, breast, and prostate cancer (Cook et al., 2014) (Table 1). In gastric cancer, Vav2/3 expression and activities were related to the degree of tumor differentiation, lymph node metastasis, and higher grade invasive and metastatic tumors (Tan et al., 2017a). Vav2 is also activated in papillary thyroid cancer, being phosphorylated through EphB3 tyrosine kinase receptors (Li et al., 2017).

In breast cancer, Vav2 activates Rac1/3 to regulate actin polymerization, invadopodia extension, matrix degradation, vesicular trafficking, and invasion (Donnelly et al., 2017; Rosenberg et al., 2017). In triple negative breast cancer cell lines, Vav2 was shown to interact with NEDD9, a scaffolding protein involved in cell migration and thus, activate Rac1 in breast cancer cell lines (Jones et al., 2017). Moreover, Vav2 was also shown to activate Rac1 and cell proliferation via its interaction with Mammalian STE20-like kinase 3, which is overexpressed in breast tumors (Cho et al., 2016). In breast cancer specimens, Vav2 expression was significantly higher in invasive cancer tissues compared to ductal carcinoma *in situ* or normal breast tissue (Jiang et al., 2014). Therefore, Vav2 is a central regulator of breast cancer metastasis.

Similar to Vav2, Vav3 expression has been associated with multiple epithelial cancers (Table 1). Vav3 expression in gastric cancer tissues was related to tumor differentiation, tumor invasion, lymphatic metastasis, neurovascular invasion and clinicopathological stage (Lin et al., 2012b; Tan, 2014; Tan et al., 2017b). Vav3 is also present in breast cancer, associating with poorly differentiated lesions. In breast cancer cells, Vav3 activated estrogen receptor (ER) α partially via PI3K-Akt signaling and promoted cell growth (Chen et al., 2015). However, Vav3 expression was higher in ER negative tissue, and high levels of nuclear Vav3 was associated with poorer endocrine therapy response (Aguilar et al., 2014). Similarly, in colorectal cancer, Vav3 expression was higher in malignant tissue compared to normal tissue, correlating with invasion and proliferation, and thus an advanced stage with poorer prognosis (Uen et al., 2015). Vav3 has been extensively studied in prostate cancer, being detected in prostate and androgen-independent prostate cancer cells. During androgen deprivation, Vav3 expression was induced and increased in LNCaP prostate cancer cells activating PI3K-Akt signaling, similar to its action in breast cancer (Hirai et al., 2014). Therefore, Vav3 overexpression may contribute to androgen receptor (AR) signaling through the PI3K-Akt pathway to stimulate cancer cell growth (Dong et al., 2006). Consequently, higher Vav3 expression was correlated with prostate cancer metastasis and recurrence. Several studies have also shown that Vav3 is activated by the tyrosine kinase EphA2 receptor to activate Rac and thus, increase cancer cell migration and proliferation (Lin et al., 2012a). Therefore, Vav3 appears to be activated by multiple signaling mechanisms to converge on Rac activation in metastatic cancer.

Structural and mutational analysis of the Vav1 DH-PH-CRD domain interaction with Rac1 revealed unique interactions of the Vav1 DH domain with the switch I and II regions of Rac1 (Chrencik et al., 2008). Accordingly, EHop-016, a Rac inhibitor developed by our group as a structural derivative of NSC23766, unlike NSC23766, which interacts with Tryp56 in the Switch II region and inhibits Tiam-1 and Trio, interacts with Val36 in the Switch I region and Asn 39 in the Switch II region of Rac, *in silico* (Montalvo-Ortiz et al., 2012). These residues have been shown to interact tightly with Glu201 and Gln331 in the DH domain of Vav (Chrencik et al., 2008). Accordingly, EHop-016 blocked the interaction between Vav1/2 and Rac with an IC_50_ of ∼1 μM in metastatic breast cancer cells. EHop-016 also reduced mammary tumor growth, angiogenesis, and metastasis *in vivo* (Hernández et al., 2010; Montalvo-Ortiz et al., 2012; Dharmawardhane et al., 2013; Castillo-Pichardo et al., 2014). Subsequently, EHop-016 was demonstrated to be effective in other cancer types, such as prostate, leukemia, melanoma, T lymphocytes, and fibrosarcoma (Montalvo-Ortiz et al., 2012; Manes and Pober, 2013; Martin et al., 2013; Maes et al., 2014; Okada et al., 2017; Chen et al., 2019). Moreover, EHop-016 was recently shown to revert cisplatin resistance in esophageal squamous cell carcinoma *in vivo* and *in vitro*, further validating its therapeutic applicability (Schmit et al., 2019). Vlaar et al. (2018) recently synthesized a series of EHop-016 derivatives in an attempt to improve its potency further. Specifically, the compounds 3b and 11b were described, where compound 11b was four times more potent than EHop-016 in inhibiting metastasis. Another derivative of EHop-016 that was recently described by us, is the small molecule MBQ-167, a dual Rac/Cdc42 inhibitor, which is ten times more potent than its predecessor (Humphries-Bickley et al., 2017). Nonetheless, the specific mechanism by which this compound interacts with Rac and Cdc42 GTPases has yet to be described in detail.

Another VAV family targeting drug is Azathioprine, an immunosuppressive drug that has been shown to inhibit Vav1 signaling in immune cells. In a recent study, azathioprine also blocked Vav1 signaling in pancreatic tumor cells, inhibiting cell migration, and decreasing metastasis in mouse models (Razidlo et al., 2015). The metabolite of azathioprine, 6-Thio-GTP, also inhibits Vav-mediated activation of Rac1, as reported from breast cancer cells and lymphocytes (Poppe et al., 2006; Menna et al., 2009).

### Targeting P-Rex

P-Rex1 was first identified in neutrophils as a GEF unique for Rac1, even though it can activate Rac1,2,3, RhoG, Cdc42, and TC10 (Jaiswal et al., 2013). P-Rex1 is composed of a DH, PH, 2 DEP, PDZ, and an inositol polyphosphate 4-phosphatase homology domain, and is thus, activated by phosphatidylinositol 3,4,5 trisphosphate (PIP_3_) through the PH domain, and by Gβγ via the DH domain. P-Rex activity is also regulated by a negative phosphorylation of the DEP domain by protein kinase A (PKA), which results in an autoinhibitory interaction with the DH/PH domains (Kazanietz et al., 2018). PAK has also been shown to phosphorylate P-Rex1 and P-Rex2, decreasing its GEF activity in a feedback inhibition manner (Barrows et al., 2015; Barrows et al., 2016). Thus, phosphorylation patterns in both P-Rex1 and 2 regulate Rac1 activation and has potential for designing novel targeted therapies (Mayeenuddin and Garrison, 2006; Montero et al., 2016).

Both P-Rex1 and P-Rex2, possess oncogenic activities, and are expressed and mutated in multiple cancer types (Srijakotre et al., 2017) (Table 1). P-Rex1 is activated by human epidermal growth factor receptor (HER2) and GPCRs, such as the chemokine receptor CXCR4 in breast cancer, and overexpressed in ER and HER2 positive luminal A and B breast cancer tissues compared to normal breast tissue. P-Rex1 expression was actually low in triple-negative primary tumors but was upregulated in distant metastases (Marotti et al., 2017; Casado-Medrano et al., 2018; Kazanietz et al., 2018). Furthermore, demethylation in the PREX promoter has been associated with breast cancer mortality (Montero et al., 2011). However, recent data using MMTV-P-Rex1 transgenic mice have shown that P-Rex1 is not necessary for mitogenesis or survival in breast cancer, thus indicting that it may be active in late stage metastases, as has been shown by microarray analysis of breast cancer cells where P-Rex1 expression was associated with matrix metalloproteinase (MMP-10) expression, and thus, a more invasive phenotype (Barrio-Real et al., 2016).

The P-Rex2 isoform P-Rex2a, is an inhibitor of the PTEN tumor suppressor, consequently activating the PI3-Kinase pathway, and thus cancer malignancy (Leslie, 2009). In endometrial tumors tissues, P-Rex2a was associated with poor prognosis independent of PTEN expression status (Takeshita et al., 2018). P-Rex2 mutations are also reported from metastatic melanoma, which results in a truncated protein, losing its ability to bind to PTEN and increasing its GEF activity (Berger et al., 2012; Deribe, 2016; Lissanu et al., 2016).

Therefore, inhibiting P-Rex may block breast cancer metastasis, and the compound 1A-116, an analog of the previously discussed ZINC69391, was shown to inhibit the interaction of the GEF P-Rex1 with Rac1 with an IC_50_ = 4 μM. IA-116 demonstrated higher antiproliferative potency than its parental compound in metastatic breast cancer cells, and decreased metastasis to the lung by 60% in an *in vivo* model of experimental metastasis (Cardama et al., 2014a). Also, IA-116 reduced cell proliferation and invasion in other cancer types, such as leukemia and glioma (Bouquier et al., 2009; Cardama et al., 2014b). In another study, treatment with 1A-116 reverted therapy resistance to tamoxifen in breast cancer cells (Gonzalez et al., 2017), demonstrating the therapeutic potential of Rac inhibitors in overcoming therapy resistance.

### Targeting FYVE, RhoGEF and PH Domain-Containing Protein or Faciogenital Dysplasia Protein (Fgd)

From the human FYVE domain containing proteins, those within the Fgd subfamily act as Rac/Cdc42 GEFs due to their DH domain and two PH domains (Eitzen et al., 2019). Recently, biochemical analysis of Fgd5, a member of the Fgd family of proteins, showed specificity for Rac1 activation (Park et al., 2019), even though FGD1 has been described as a GEF for Cdc42 (Egorov et al., 2009). Fgd5 has a DH domain similar to Trio with preferential activation of Rac1, and has been related to poorer prognosis in breast cancer patients (Valla et al., 2017). Park et al. (2019) identified the small molecule aurintricarboxylic acid through a surface plasmon resonance screening as a compound that binds to Fgd5, thus inactivating Rac1 with an IC_50_ of 157 nM. Further studies are needed to validate these results *in vivo* and to delineate the utility of targeting Fgd5 to block cancer progression.

### Targeting DOCKs

One of the most commonly known oncogenic GEFs from the DOCK family is DOCK1 (also known as DOCK180), a Rac specific GEF. In addition to DHR1 lipid binding and catalytic DHR2 domains, DOCK1 also contains a SH3 domain and a proline rich motif. The GEF function of DOCK1 is activated by binding to engulfment and cell motility protein 1 (ELMO) through its SH3 domain, as well as phosphorylation. DOCKs are activated by oncogenic RTKs, where in glioblastoma, the oncogenic epidermal growth factor receptor (EGFRvIII) and platelet derived growth factor receptor (PDGFR) induced Src-mediated tyrosine phosphorylation of DOCK1 to activate Rac1 and promote cell migration and invasion (Feng et al., 2011, 2012). PDGFRα mediated protein kinase A action was shown to activate DOCK1 via serine phosphorylation (Feng et al., 2011, 2015). Moreover, in a HER2+ breast cancer mouse model, DOCK1 promoted invasion to the lungs, where the lung metastases overexpressed DOCK1 compared to primary tumors (Laurin et al., 2013). DOCK1 expression and mutations have been reported from a number of cancers (Table 1).

Such evidence of an oncogenic and metastasis promoting role for DOCK family members has led to an interest in the development of DOCK inhibitors. TBOPP, a DOCK1 selective inhibitor, inhibited tumor growth and metastasis in mice with lung carcinoma tumors (Tajiri et al., 2017). This compound also targeted DOCK1 in melanoma cells with the oncogenic Rac1 P29S mutation (Tomino et al., 2018). Hence, DOCK1 inhibition could be an approach for the treatment of cancers associated with the Rac1 P29S oncogenic mutation.

DOCK2, is expressed in hematopoietic cells and has been implicated in a number of inflammatory diseases and cancer (Chen et al., 2018). Therefore, DOCK2 is considered to be a viable drug target for leukemia, where DOCK2 is overexpressed in chronic lymphocytic leukemia (Wu et al., 2017; Hasan et al., 2018).

The small molecule CPYPP was identified as a DOCK2-inhibitor that binds to its DHR-2 domain (Nishikimi et al., 2012). Nonetheless, the utility of this compound is limited by its weak potency (IC_50_ = 23 *μ*M for DOCK2) and non-specificity, since it also inhibits the GEFs DOCK5 and Trio (Ferrandez et al., 2017), which could lead to unwanted side effects.

On the other hand, a naturally occurring DOCK2 inhibitor is cholesterol sulfate, which also binds to the catalytic domain of DOCK2 but at a higher potency *in vitro* (IC_50_ = 2 *μ*M). Cholesterol sulfate limited inflammation by inhibiting leukocyte migration and Rac activation in murine T-cells, an effect not observed with other cholesterol derivatives (Sakurai et al., 2018). However, the therapeutic efficacy and specificity of this compound in cancer models remains to be elucidated.

Recent efforts have been directed toward the development of DOCK2 inhibitory peptides conjugated with different moieties to allow cell permeability. Adachi et al. (2017) produced various cell-penetrating peptides (CPP) that were conjugated with a partial sequence of influenza A viral protein (PB1-F2) to increase cell permeability. Inhibitory activity of these peptides to DOCK2 was in the nanomolar range, and conjugation with PB1-F2 increased cellular uptake of these peptides (Adachi et al., 2017). Other DOCK2-selective inhibitory peptides are Dcpep-3 and Dcpep-4 (Sakamoto et al., 2017). Dcep-4 has an IC_50_ of 6 nM for Rac1 and could be valuable in the treatment of leukemia and autoimmune diseases. Nonetheless, variable absorption and lack of specific localization could prove to be a challenge in the development of these peptides as therapeutics. Additionally, *in vivo* efficacy of cell-penetrating peptides remains to be demonstrated.

Another activator of Rac1 that is also an attractive target of the DOCK family is DOCK5. DOCK5 has also being implicated in acute myeloid leukemia (Biswas et al., 2019, 30668141), and recently, an oncogenic variant of DOCK5 was described from head and neck squamous cell carcinoma (Liu et al., 2018).

Vives et al. (2011) discovered the small molecule, C21, as an inhibitor of DOCK5 through screening, using a yeast-based assay. This molecule targets the DHR2 domain of DOCK5, causing an abortive conformation that results in non-competitive inhibition (Ferrandez et al., 2017). In models of bone metastasis, C21 was able to protect mice against bone degradation without causing side effects, which makes it a potential therapeutic against osteolytic diseases (Vives et al., 2015).

### Inhibitors of the Cdc42-GEF Interaction

#### Targeting [Dbl’s Big Sister (Dbs)]

Dbs is another DH family member that is ubiquitously expressed and was identified as an oncogene by its ability to transform NIH3T3 fibroblasts (Whitehead et al., 1995). Full-length Dbs encompasses 1149 amino acids and contains, in addition to the DH and PH domains, a putative N-terminal Sec14 domain, two spectrin-like repeats and a C-terminal SH3 domain. Structural studies of the DH/PH domains of Dbs complexed with Cdc42 revealed that the binding interactions were unique and distinct from those described for Tiam-1 and Trio (Rossman et al., 2002). Even though Dbs was first described as an activator of RhoA and Cdc42, but not Rac1, in the activation of multiple signaling pathways using specific inhibitors (Whitehead et al., 1999; Snyder et al., 2002), a subsequent study using siRNA directed at Cdc42 and Rac1 demonstrated a role for both Cdc42 and Rac1 in activating breast cancer cell migration. However, in this study, oncogenic Dbs demonstrated increased Rho activation, and thus, even though Rho activity may not be relevant for the demonstrated Rac/Cdc42 mediated breast cancer cell migration via downstream activation of focal adhesion kinase/p130Crk associated substrate, the enhanced Rho activity in oncogenic Dbs expressing breast cancer cells may still contribute to other cancer promoting signaling pathways in cancer (Liu et al., 2009).

AZA197 is a small-molecule inhibitor of the Cdc42-Dbs interaction that was shown to inhibit cancer cell proliferation and invasion with an IC_50_ of 1–10 *μ*M. AZA197 also reduced tumor growth and extended survival in a mouse model of colon cancer (Zins et al., 2013). This molecule was identified through an *in vitro* screen of compounds similar to the previously discussed Rac-inhibitor NSC23766. Nonetheless, AZA197 showed toxicity in concentrations higher than 20 *μ*M in NIH3T3 fibroblasts and colon cancer cells, and may not prove to be pharmacologically useful.

Another molecule that inhibits Cdc42 activation through Dbs is Compound 19, an α-helix mimetic that targets the K774, L777, and Q770 residues of Dbs that mediate the interaction between Dbs and Cdc42 (Cummings et al., 2009). However, the therapeutic applicability of Compound 19 in cancer remains to be tested. Additionally, its high IC_50_ (IC_50_ = 67 *μ*M) limits its therapeutic potential.

### Targeting Intersectin (ITSN)

ITSN is another DH family member, which primarily activates Cdc42 to regulate the actin cytoskeleton (Humphries et al., 2014). ITSNs are multidomain adaptor proteins regulating endocytosis and cell signaling via RTK/PI3-K pathways, and have been implicated in neurodegenerative diseases and cancer. Structurally, N-terminal Eps15 homology (EH1 and EH2) domains, a coiled coil region, and five SH3 domains are present in the short isoform (ITSN-S), while the long isoform of ITSN (ITSN-L) has an additional C-terminal DH/PH domain, which confers GEF activity (Hunter et al., 2013). Even though low expression of ITSN1 was recently correlated with higher grade in breast and lung cancer (Xie et al., 2019; Zhang and Zhang, 2019), A role for ITSN in neuroblastoma has been shown by a study where knockdown of ITSN decreased orthotopic neuroblastoma growth in mice (Harris et al., 2017).

So far, four small molecules have shown to block the interaction of Cdc42 with ITSN; these are Pirl1, CASIN, ZCL278, and ZCL367. The compound Pirl1 was developed to target the Phe56 residue in Cdc42, which has been demonstrated to be crucial for GEF binding. This molecule inhibits the PIP2-mediated GEF activity of the Cdc42/RhoGDI complex (Peterson et al., 2006). A derivative of Pirl1, CASIN (Cdc42 activity-specific inhibitor), demonstrated *in vitro* and *in vivo* efficacy in colorectal cancer models with an IC_50_ of 2 *μ*M (Sakamori et al., 2014). CASIN could also prove to be useful in improving hematopoietic stem cell mobilization and benefiting bone marrow transplant recipients (Liu et al., 2019). ZCL278 is another ITSN inhibitor, identified through virtual screening to directly bind Cdc42 by interacting with several residues of its Switch I region, essential for the interaction with ITSN. ZCL278 blocked prostate cancer cell migration and may have potential as a metastatic prostate cancer therapeutic (Friesland et al., 2013).

Recently, Aguilar et al., 2019 developed a potent inhibitor of the Cdc42-ITSN interaction, named ZCL367. With an IC_50_ of 0.098 nM, ZCL367 demonstrated beneficial effects in lung and prostate cancer cells by inhibiting migration, proliferation, and cell cycle progression. Its anticancer potential was further validated in a xenograft mouse model of lung cancer, where it decreased tumor growth. This molecule also inhibits Rac1 but, to a lesser degree (IC_50_ = 0.19 nM) (Aguilar et al., 2019). Hence, ZCL367’s efficacy and low IC_50_ makes it a promising anti metastatic cancer therapeutic for further development.

## Rac/Cdc42 Inhibitors in Therapy Resistance

So far, no Rac/Cdc42 inhibitors have received FDA approval for clinical trials; however, burgeoning evidence has positioned Rac and Cdc42 as ideal targets for anti-metastatic cancer therapy. Rac1 and Cdc42 are unique in that they act as molecular switches that do not have to be mutated to drive cancer progression but are activated by oncogenic cell surface receptors. These receptors include RTKs, GPCRs, and integrins, which signal to other oncogenes such as PI3-K and Ras that can in turn activate Rac/Cdc42 GEFs, which themselves can be oncogenic (Figure 1). Therefore, upregulated Rac and Cdc42 can drive malignant signaling during acquisition of resistance to cancer therapies targeting cell surface receptors, by acting downstream of therapy resistance mechanisms such as Ras/MAPK and PI3-K/mTOR signaling (Baker et al., 2014; Cardama et al., 2018; Maldonado and Dharmawardhane, 2018; De et al., 2019).

Numerous studies have implicated Rac/PAK activities with the maintenance of mesenchymal stem cell-like populations in epithelial cancers; and thus, therapy resistance (Zhao et al., 2011; Ong et al., 2013; Zhu et al., 2015; Goel et al., 2016; Huynh et al., 2016; Aboukameel et al., 2017; Lai et al., 2017; Morrison Joly et al., 2017; Cardama et al., 2018; Goka et al., 2019). Specifically in breast cancer, Rac/Cdc42/PAK signaling is implicated with therapy resistance of HER2-type (Wang et al., 2006; Ebi et al., 2013; Laurin et al., 2013; Dokmanovic et al., 2014; Desai et al., 2016; Hampsch et al., 2017), triple negative (De et al., 2017), and ER(+) cancers (Cai et al., 2003; Gonzalez et al., 2017). TCGA data show that Rac1 or PAK1 overexpression is associated with malignant breast cancer and significantly diminishes HER2 type patient survival within 10 years following diagnosis (Fang et al., 2016). Accordingly, Rac/PAK inhibitors have the potential to overcome resistance to HER2/EGFR targeted therapies in epithelial cancers that overexpress these receptors.

The success of current oncogenic receptor targeted therapies for high grade cancers continue to be marred by acquired and intrinsic therapy resistance due to activation of downstream signaling. Therefore, a viable strategy to mitigate HER2/EGFR therapy resistance is combination therapy with other therapeutics targeting the downstream signaling pathways (Vrbic et al., 2013). Of the 20% of invasive HER2 type breast cancers that are treated with HER2 targeted therapeutics, such as trastuzumab (Herceptin), the monoclonal antibody targeting HER2, ∼25% of patients eventually develop resistance. Trastuzumab resistant breast cancers circumvent HER2 inhibition via bypass signaling and activation of downstream pathways independent of HER2 (Normanno et al., 2006; Bender and Nahta, 2008; Hynes and MacDonald, 2009; Weigelt et al., 2010; Wilken and Maihle, 2010; Brünner-Kubath et al., 2011; Nahta, 2012; Vu and Claret, 2012; Wong and Lee, 2012). Notably, a number of HER2 therapy resistance signaling pathways (Vu and Claret, 2012) are regulated by Rac signaling and targeting Rac has been shown to block breast cancer metastasis (Bracho-Valdés et al., 2011; Saci et al., 2011; Katz et al., 2012). Studies have shown that Rac1 activation is critical for resistance to trastuzumab in HER2+ breast cancer cells with upregulated insulin growth factor receptor (IGFR) (Zhao et al., 2011). Our data using a trastuzumab resistant HER2 type metastatic cancer have demonstrated the efficacy of Rac/Cdc42 inhibitors to block metastatic cancer cell functions, tumor growth, and metastasis (Montalvo-Ortiz et al., 2012; Dharmawardhane et al., 2013; Castillo-Pichardo et al., 2014); thus highlighting the utility of Rac inhibitors to overcome trastuzumab resistance.

Therapy with the next generation small molecule EGFR/HER2 inhibitor lapatinib also results in resistance. Similar to trastuzumab, lapatinib resistance circumvents its kinase inhibitory function by acquiring point mutations in HER2 and EGFR, as well as via elevated downstream signaling (Weigelt et al., 2010; Wilken and Maihle, 2010; Zuo et al., 2016; Xu X. et al., 2017). Rac1 has been shown to be overexpressed in lapatinib-resistant HER2 type breast cancer and is considered to be a viable therapeutic for sensitization of lapatinib resistant tumors (Wetterskog et al., 2014). In addition, Rac1 inhibition has been shown to overcome gefitinib (EGFR-targeted therapeutic) resistance in NSCLC (Kaneto et al., 2014). Recent studies with glioblastoma cells also demonstrate the potential of the Tiam-1/Trio/Rac inhibitor NSC23766 in combination therapy with eroltinib, another tyrosine kinase inhibitor (Karpel-Massler et al., 2017). Moreover, EHop-016, a Vav/Rac inhibitor developed by us, was recently shown to overcome resistance by combined cancer therapy with Akt/mTOR inhibitors (Okada et al., 2017). Thus, Rac inhibitors are poised for dual therapy with HER2/EGFR or Akt/mTOR targeting therapies.

In addition, Rac/Cdc42 inhibition has the potential to sensitize cancers to current chemotherapies such as taxanes and anthracyclines. Since cytotoxic cancer therapeutics are designed to kill actively dividing cells, this can lead to selection of non-dividing stem cells that still have the capacity to migrate and establish tumors at distant sites. Several studies have implicated Rac and Cdc42 signaling in stem cell maintenance through activation of Wnt, Notch, and YAP signaling in breast cancer, NSCLC, and lymphomas (De et al., 2017; Hicks-Berthet and Varelas, 2017; Lai et al., 2017; Olabi et al., 2018; Katoh and Katoh, 2020). Therefore, as has been shown by us using mammosphere assays, Rac/Cdc42 inhibition can reduce stem cell-like growth in breast cancer (Humphries-Bickley et al., 2017).

Recent studies on metastasis show that chemotherapy may also affect multicellular interactions in the tumor microenvironment for metastasis (TMEM) by recruiting macrophages, mesenchymal stem cells, platelets, etc., which increases cancer cell survival, invasion, angiogenesis, and metastasis (Karagiannis et al., 2018). Given the central role of Rac and Cdc42 in immune cell function, Rac/Cdc42 inhibitors also have potential for targeting immune cell migration and inflammation, including infiltration of perivascular macrophages, increased vascular permeability, and lymphatic penetration (Infante and Ridley, 2013; Biro et al., 2014; Durand-Onaylı et al., 2018). Indeed, we have shown that Rac inhibition decreases macrophage and neutrophil penetration of the TMEM (Castillo-Pichardo et al., 2014; Humphries-Bickley et al., 2017). Therefore, an additional benefit of Rac/Cdc42 inhibition is the reduction in bone marrow-derived cells that promote the metastatic dissemination of cancer cells in the TMEM.

Accordingly, recent studies have also implicated a role for Rac/Cdc42 inhibitors in combination with cytotoxic therapies for maximum efficacy. In esophageal squamous cell carcinoma, Rac1 expression was associated with poor prognosis and therapy resistance. Use of the Vav/Rac inhibitor EHop-016 was shown to overcome cisplatin resistance in esophageal squamous cell carcinoma *in vitro* and *in vivo* and by decreasing Akt/FOXO3a signaling and glycolysis (Schmit et al., 2019; Zeng et al., 2019), further validating its therapeutic applicability. Moreover, Tiam1 and Rac1 were overexpressed in a 3-D model of lymphoma, where treatment with Tiam1/Rac1 inhibitor NSC23766 overcame chemoresistance to doxorubicin (Ikram et al., 2018). NSC23766 has also been shown to overcome resistance to fludarabine in chronic lymphocytic leukemia where Tiam1/Rac1 is upregulated (Hofbauer et al., 2014). Also, Rac1 inhibition with NSC23766 increases the cytotoxic effect of Adriamycin, in the treatment of mantle cell lymphoma, an aggressive B-cell lymphoma (Tian et al., 2018). Therefore, combination therapy studies with standard cytotoxic agents further supports the clinical significance of targeting Rac and Cdc42.

## Conclusion

Current studies on targeting Rac and Cdc42 have identified compounds that are poised to emerge as leading antimetastatic drugs for combination therapy with cytotoxic cancer therapies and to overcome resistance to current therapies targeting RTKs and PI3-K/Akt/mTOR signaling (Figure 1). Due to the specificity of their interaction with different oncogenic GEFs, drugs that block the Rac/Cdc42-GEF interaction need to be prudently targeted to different tissues and types of cancer according to their expression and activation of specific GEFs. However, there is a pressing need to improve the efficacy of the currently available GEF targeting drugs and demonstrate *in vivo* activity at physiologically relevant concentrations, prior to their translational development.

In addition, there is also a need to target multiple GEFs, such as the DH family GEF Ect2. Ect2 is a proto-oncogene with GEF activity toward Rho, Rac, and Cdc42, and thus regulates cell division, polarity, and invasion/migration (Tatsumoto et al., 1999). Ect2 has been shown to be overexpressed in multiple cancers and associated with poor survival (Saito et al., 2004; Justilien and Fields, 2009; Fields and Justilien, 2010; Iyoda et al., 2010; Huff et al., 2013; Wang et al., 2016, 2018; Guo et al., 2017; Bai et al., 2018) (Table 1). Inhibitors targeting Ect2 have yet to be identified, but it is imperative to develop specific inhibitors to block the activation of Rho GTPases by Ect2 in cancer cells.

Overall, this review provides ample evidence for the utility of Rac/Cdc42 inhibitors both individually, and in combination with current targeted and cytotoxic therapeutics to overcome therapy resistance and augment established cancer therapies to specifically block metastasis. In addition to combined therapy with existing cancer therapeutics, cocktails of Rac/Cdc42 GEF targeting compounds may also be developed for maximum therapeutic efficacy. In conclusion, the plethora of compelling data discussed in this review realizes the promise of Rac/Cdc42 inhibitors as viable therapeutics for metastatic cancer, and we look forward to their routine use in standard cancer care.

## Author Contributions

MM, JM, and SD wrote the manuscript. MM prepared the Table 2. JM prepared the Table 1. LV prepared the Figure 1.

## Conflict of Interest

The authors declare that the research was conducted in the absence of any commercial or financial relationships that could be construed as a potential conflict of interest.
